# Unraveling the Roles of HIF-1, HO-1, GLUT-1 and GLUT-4 in Myocardial Protection

**DOI:** 10.53941/ijddp.2024.100016

**Published:** 2024-08-26

**Authors:** Lionel Chong, Nicholas Dushaj, Ani Rakoubian, Johnathan Yarbro, Satoru Kobayashi, Qiangrong Liang

**Affiliations:** Department of Biomedical Sciences, New York Institute of Technology, College of Osteopathic Medicine, Old Westbury, NY 11568-8000, USA

**Keywords:** HIF-1, HO-1, GLUT-1, GLUT-4, cardioprotection

## Abstract

Cardiomyocytes are highly dependent on oxygen for optimal function. Disruption of oxygen availability, as in the case of ischemic heart disease, can significantly impair heart function. Moreover, comorbidities like diabetes, hyperlipidemia, and hypertension can exacerbate ischemic cardiac injury. However, cardiomyocytes possess inherent protective mechanisms that can be activated to enhance myocardial survival under such conditions. Understanding the functions and regulatory mechanisms of these cardioprotective genes is crucial for advancing our knowledge of cardiovascular health and for developing therapeutic strategies. This review examines the intricate mechanisms of cardioprotection, with a focus on key genes and proteins, including hypoxia-inducible factor-1 (HIF-1), heme oxygenase-1 (HO-1), glucose transporter 1 (GLUT-1), and GLUT-4. In addition, the review explores the roles and regulation of these factors in the heart under ischemic stress, shedding light on their relevance in conditions like diabetes, hypertension, and hyperlipidemia/atherosclerosis. Moreover, it highlights the complex interplay among their mechanisms and suggests opportunities for developing targeted therapiesfor the treatment of ischemic heart disease, hypertension, and hyperlipidemia.

## 1. Introduction

Cardiomyocytes homeostasis is highly dependent on oxygen availability, and thus adequate oxygen levels (normoxia) are crucial for cardiomyocyte function. However, various factors contribute to hypoxic conditions, with ischemic heart disease being one of the most prominent causes. Prolonged exposure to hypoxia is detrimental to cardiomyocytes and can impair heart function.

Despite the challenges posed by ischemic stress, cardiomyocytes possess endogenous protective mechanisms that are activated under such conditions, collectively referred to as cardioprotection. This intricate process involves a network of genes and proteins, with numerous points of regulation, responsible for executing various cellular cardioprotective mechanisms. Among these, hypoxia-inducible factor-1 (HIF-1), heme oxygenase-1 (HO-1), glucose transporter 1 (GLUT-1), and GLUT-4 stand out as significant protein complexes that promote cardiomyocyte survival and prevent vasculitis, a critical aspect of overall cardiovascular health.

In this review, we aim to describe the roles, regulation, and interactions of these cardioprotective genes—HIF-1, HO-1, GLUT-1, and GLUT-4. By examining their functions and regulatory mechanisms, particularly in the context of cardioprotection during ischemic stress, including conditions of ischemia or hypoxia commonly seen in cardiovascular diseases, we aim to expand our understanding of these protein complexes. Furthermore, we endeavor to provide insights into cardioprotective mechanisms under conditions such as hypertension, diabetes, and hyperlipidemia-induced stress, which often exacerbate ischemic episodes. We will also explore alternative pathways for cardiomyocyte energy generation, considering the impact of diabetes and hyperlipidemia on cellular metabolism. The current research on potential drug therapies for managing heart disease induced by diabetes and hyperlipidemia are also reviewed to reveal new therapeutic strategies for targeting these coexisting conditions.

Our focus on these genes stems from their significant roles in preventing various medical conditions, including hypertension, diabetes, and hyperlipidemia. By unraveling the multifaceted mechanisms underlying cardioprotection, we aim to contribute to the advancement of medical therapy for patients with various cardiovascular complications.

## 2. Hypoxia-Inducible Factor: A Master Regulator of Myocardial Protection

Hypoxia-inducible factor (HIF-1) is a heterodimeric transcription factor composed of two subunits: oxygen-regulated α -subunit (HIF- α) situated in the cytoplasm and the constitutively expressed β -subunit (HIF-β), also known as arylhydrocarbon receptor nuclear translocator (ARNT), located in the nucleus. There are three isoforms of HIF-α, namely, HIF-1α, HIF-2α and HIF-3α, among which HIF-1α is most-extensively studied and understood [1]. Both subunits share a basic helix-loop-helix structure, which is pivotal for sensing cellular oxygen levels and detecting hypoxia. HIF-1 regulates oxygen homeostasis by controlling oxygen supply (angiogenesis and vascular remodeling) and oxygen utilization (glucose metabolism and redox homeostasis) in the heart and other tissues [2].

**Figure 1.**
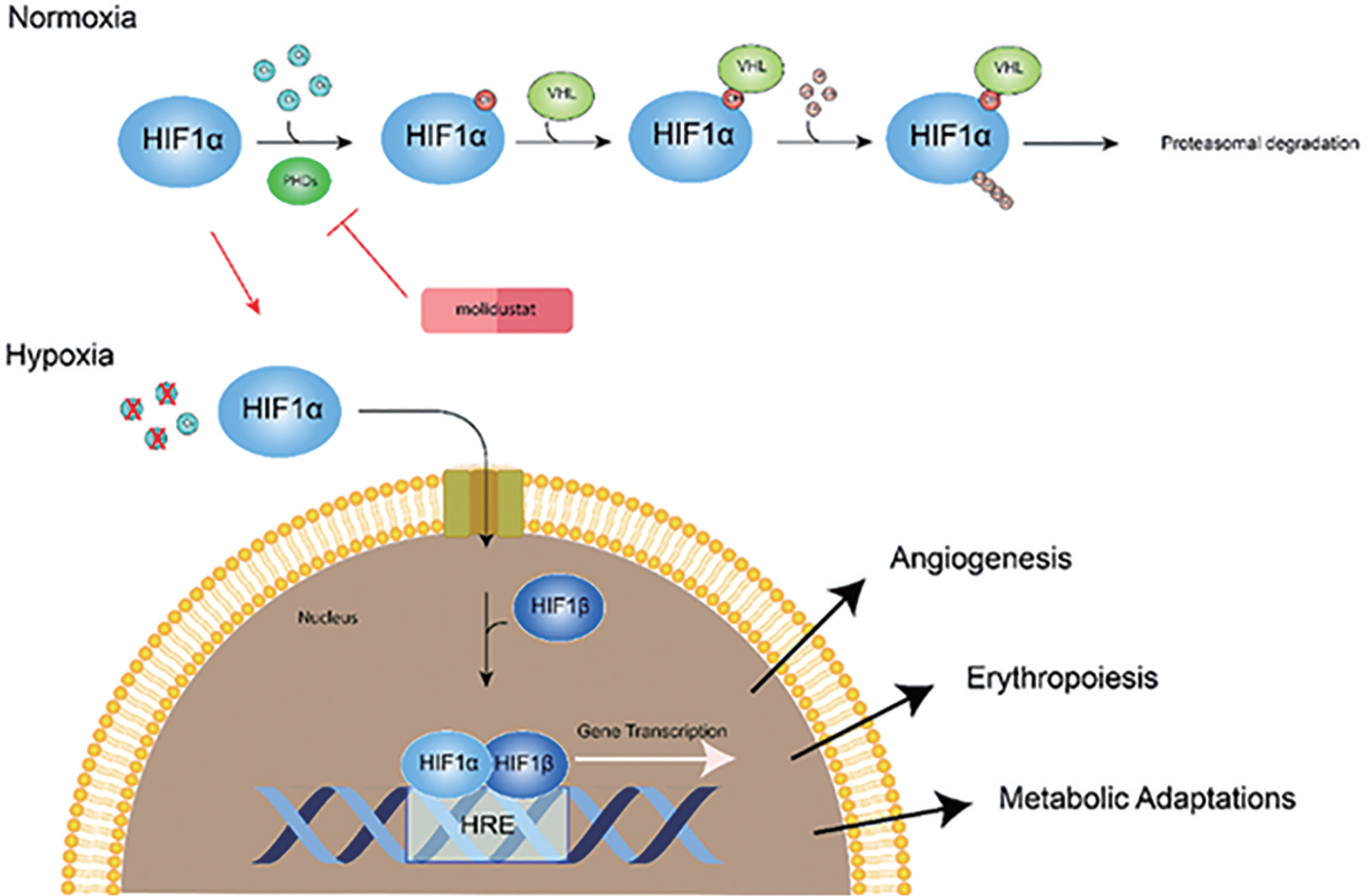
Hypoxia inducible factor 1 (HIF1) regulation during normoxic and hypoxic conditions. During normoxic conditions, HIF-1α is destabilized by proline hydroxylases (PHDs) leading to rapid degradation via the VHL-mediated ubiquitin protease pathway. Under hypoxic conditions HIF-1α is translocated to the nucleus where it forms a heterodimer with HIF-1β, which then binds to the hypoxia responsive element (HRE) sequences within the regulatory region of target genes, inducing gene transcription. Molidustat can reduce HIF-1α degradation by inhibiting PHDs.

In normoxic conditions, HIF-1α is destabilized by post-translational hydroxylation under the catalysis of prolyl hydroxylase domain enzymes (PHD1, PHD2, and PHD3), leading to pVHL (von-Hippel-Lindau protein)-dependent ubiquitination and rapid proteasomal degradation of HIF-1α (Figure 1) [3]. This disrupts the transcription of genes associated with the HIF-1 protein complex. Conversely, under hypoxic conditions, the aforementioned posttranslational modifications are limited, which inhibits HIF-1α degradation, inducing its translocation from the cytoplasm to the nucleus where it heterodimerizes with HIF-1β. Activation of the HIF-1 complex enables the binding to the hypoxia responsive element (HRE) sequence within the regulatory region of target genes, thereby inducing gene transcription (Figure 1) [2]. This activates a signaling cascade comprising over 100 downstream proteins to regulate diverse cellular functions (Figure 1), including angiogenesis, erythropoiesis, iron metabolism, energy metabolism, matrix and barrier functions, proliferation and growth, differentiation and apoptosis, among others [4,5].

The role of HIF-1 in cardioprotection has been extensively documented [2,4]. Initially, this phenomenon was observed in rodents exposed to intermittent hypoxia, in which HIF-1 protected against ischemia-reperfusion injury [6]. Subsequent studies have confirmed this finding [5,7]. HIF-1 also serves as the central mediator in ischemic preconditioning (IP)-induced cardioprotection [8,9], and even partial deficiency of HIF-1α (+/−) abolishes IP-induced cardioprotection in mice [10]. Additionally, HIF-1α may serve as an early molecular marker for myocardial ischemia or infarction [11]. HIF-1 also plays an important role in the embryologic development of the heart. Loss of HIF-1α (−/−) results in various heart defects in utero such as improper formation of the heart tube, defective vascularization of the cardiac chambers, and hyperplastic myocardium, among others, which often lead to embryonic lethality [10,12]. Mechanistically, HIF-1 activates the expression of genes such as vascular endothelial growth factor (VEGF), epidermal growth factor (EGF), tissue inhibitors of metalloproteinases (TIMPs), and others, promoting angiogenesis and perfusion to meet the rising oxygen demand by cardiomyocytes. Besides aiding in oxygen delivery systemically through processes like erythropoiesis and angiogenesis, HIF-1-regulated pathways contribute to the protection of the heart from hypoxia. They modulate metabolic pathways to optimize ATP production and upregulate the expression of numerous genes, thereby supporting cardiomyocyte survival [4,13].

Research has demonstrated the involvement of the phosphoinositide-3-kinase (PI3K)/AKT pathway in HIF-1-related cardioprotective mechanisms under hypoxic conditions. AKT is integral to insulin metabolic processes, including GLUT-4 translocation to the cell membrane for glucose uptake to generate ATP. AKT also facilitates the activation of rapamycin (mTOR), crucial for cardiomyocyte growth and cell death prevention. The EGFR-PI3K-Akt-mTOR pathway enhances VEGF and endothelial nitric oxide synthase (eNOS) expression by upregulating HIF-1α. Moreover, HIF-1α activation during inflammation upregulates the levels of target genes such as VEGF, GLUT-1, metalloproteinases, β2 integrin, adenosine receptors, and chemokine receptors [12,14].

Several clinical conditions affect HIF-1 cardioprotection, with diabetes mellitus being a common concern. In diabetes, numerous tissues experience hypoxia including the heart, yet their ability to adapt to this condition is compromised due to under-activation of the HIF signaling. This deficiency arises from the decreased HIF-1α stability and function caused by high blood sugar levels and increased fatty acid concentrations [15–17]. Consequently, in response to myocardial ischemia, critical metabolic adaptations to hypoxia diminish in diabetic heart, increasing the infarct size and decreasing cardiac function [18]. Long term diabetes can directly damage the heart muscle inducing diabetic cardiomyopathy and increasing the risk of heart failure even in the absence of hypertension, coronary artery disease and other cardiac pathologies [19,20].

Current therapeutic approaches targeting HIF-1 involve the use of HIF prolyl hydroxylase domain inhibitors, which impede the degradation of HIF-1α by prolyl hydroxylase proteins via a ubiquitin-proteasomal process. This strategy has been verified by findings from studies demonstrating the effectiveness of silencing the Prolyl 4-hydroxylase-2 gene in mitigating myocardial ischemia-reperfusion injury [7]. An example of such inhibitors is molidustat, which binds to the active site of PHD to obstruct the formation of the PHD/HIF-1α complex, thereby preventing HIF-1α degradation (Figure 1) [21]. This mechanism has proven to be effective in alleviating impaired HIF-1 response to hypoxia induced by diabetic stress, indirectly stabilizing the HIF-1α protein within cardiomyocytes [22]. Furthermore, several FDA-approved drugs, including daprodustat, desidustat, molidustat, and vadadustat, are prescribed to manage anemia associated with HIF-1 dysregulation in chronic kidney disease [23]. However, prolonged activation of HIF-1α may adversely affect cardiac function [24]. A deeper understanding of HIF-1 regulation not only underscores the complexity of cardioprotective mechanisms but also reveals promising therapeutic targets for further drug development.

## 3. Heme Oxygenase-1: A Defender against Oxidative Cardiac Injury

**Figure 2.**
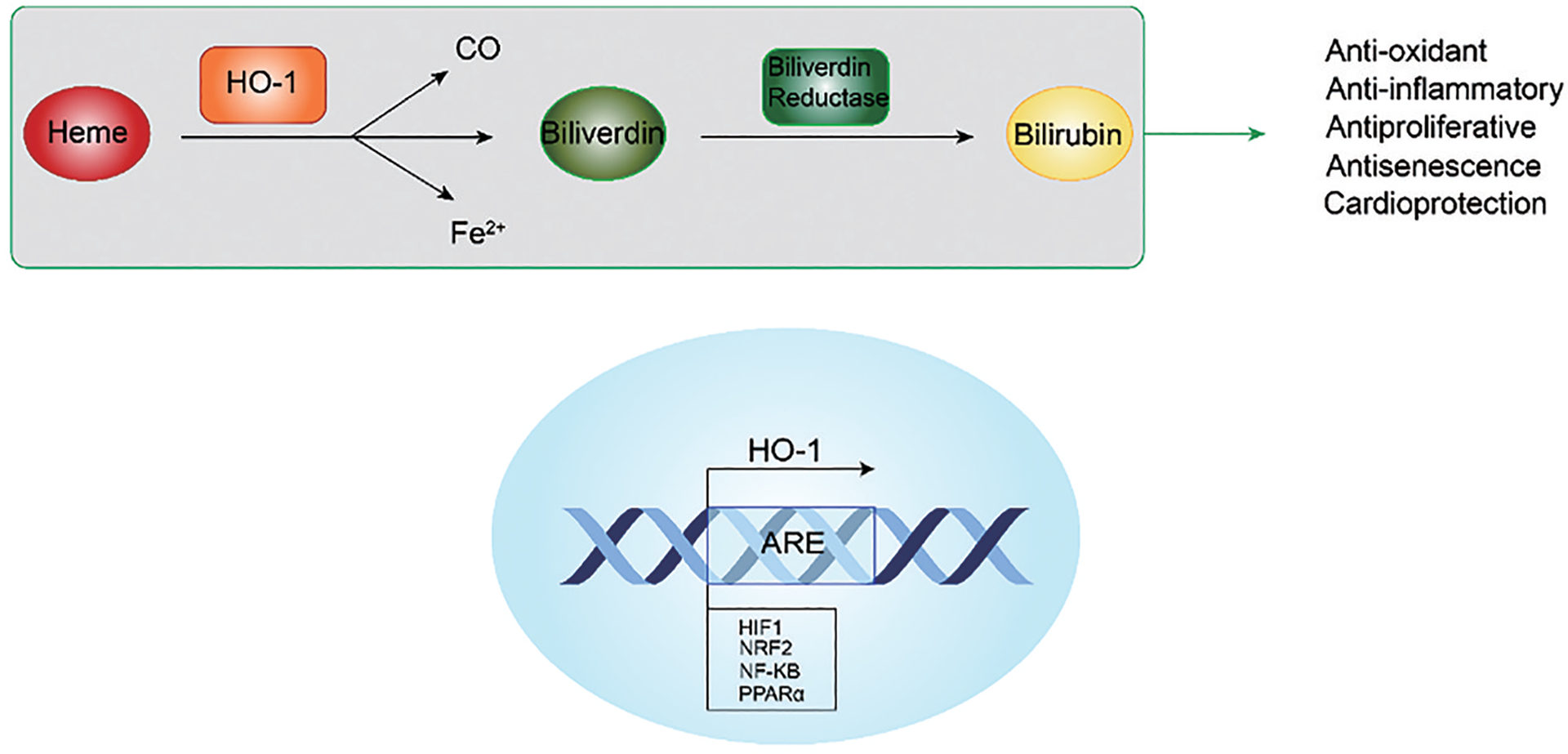
Degradation of heme via heme oxygenase-1 (HO-1). Heme is converted to biliverdin via HO-1 and releases carbon monoxide and free iron in the process. Biliverdin is then converted to bilirubin via biliverdin reductase. The products of this reaction have several beneficial effects including antioxidant, anti-inflammatory, antiproliferative, antisenescence, and cardioprotective effects. The HO-1 protein can be upregulated by several transcription factors including HIF1, NRF2, NF-KB, and PPARα, which bind to antioxidant response element (ARE) sequences within the HO-1 promoter.

Heme oxygenase (HO) is the key enzyme regulating heme catabolism. The protein has two distinct isoforms expressed in mammals, HO-1 and HO-2. While HO-2 is constitutively expressed, the expression of HO-1 is highly inducible by diverse stimuli like heme, nitric oxide, heavy metals, growth factor, cytokines, modified lipids and others [25]. In addition, HO-1 is a ubiquitous endoplasmic reticulum-anchored protein [26] that catalyzes the rate-limiting step of the degradation of heme, releasing carbon monoxide (CO), free iron, and biliverdin, which is then converted to bilirubin by biliverdin reductase (Figure 2) [27]. It was reported that HO-1 and the heme catabolic products exert many beneficial effects in the cardiovascular system [28]. Indeed, the induction of HO-1 and the ensuing production of biliverdin and bilirubin reduce adiposity and oxidative stress, decrease excessive heme levels, elevates antioxidant enzymes, and inhibit NADPH oxidase [29].

The HO-1 promoter contains antioxidant/electrophile response elements (ARE/EpRE) and other DNA sequences that facilitate the binding of various transcription factors. Under normoxic conditions, HO-1 is expressed at low basal levels. However, in hypoxic environments, such as during ischemic events, upregulation and/or activation of HIF-1 increases the expression of HO-1 transcript and protein [30], thereby reducing the production of reactive oxygen species (ROS) and mitigating ischemia/reperfusion injury in the heart [5]. Besides HIF-1, the HO-1 promoter is also activated by other transcription factors such as nuclear factor-kappa B (NF-kB), nuclear factor erythroid 2-related factor 2 (Nrf 2), and activator protein-1 (AP-1) [31]. Nrf 2 controls the expression of antioxidant-response genes, including HO-1. Nrf 2 and HIF-1 co-activate the HO-1 promoter to increase its transcription, promoting cell growth and survival (Figure 2).

Deletion of the human HO-1 gene induces damage to the liver, kidney, and vasculature system [32]. Similarly, mice lacking HO-1 exhibit elevated heme levels and a compromised stress defense system [33], underscoring the crucial role of HO-1 in maintaining organ homeostasis. Targeting HO-1 has emerged as a promising therapeutic approach for preventing the development of various cardiovascular diseases [34]. The cytoprotective properties of HO-1 and its enzymatic byproducts are well-documented [35]. Ischemia/reperfusion initiates a cascade of events leading to increased levels of cellular proteins such as troponin I, creatinine kinase MB (CK-MB), and the release of free mitochondria, heme, ATP, and mitochondrial DNA (mtDNA). The elevated levels of cellular components significantly increased HO-1 activity. HO-1 activation is considered an adaptive response that mitigates the detrimental effects of ischemia/reperfusion on cardiac tissues, which contributes to the restoration of normal physiological function and tissue integrity [35,36]. For instance, HO-1 transgenic overexpression improves heart function and mitigates cardiomyocyte senescence triggered by ischemic injury [37]. Delivery of HO-1 gene via adeno-associated virus (AAV-HO-1) prolongs cardiac allograft survival, preserves left ventricular function, and reduces mortality in mice [38]. These cardioprotective effects of AAV-HO-1 have been replicated in a porcine study [39]. Conversely, HO-1 downregulation induces organ damage in both humans and mice by enhancing oxidative stress [33,40].

Upregulation of HO-1 is also cardioprotective against ischemic-reperfusion injury in diabetes. Reduced HO-1 expression in cardiomyocytes has been linked to ventricular fibrillation in diabetic animal models [28, 41,42]. Moreover, in a diabetic mouse model induced by streptozotocin (STZ), HO-1 overexpression significantly reduced cardiac oxidative stress, inflammation, and apoptosis. This improvement was ascribed to decreased p53 expression and increased Bcl-2 expression. These findings highlight the significance of HO-1 in preventing diabetic cardiomyopathy and its potential to serve as a therapeutic target for managing cardiovascular complications linked to diabetes [43]. Indeed, the protective role of HO-1 has prompted extensive research into pharmacologically boosting its levels [29]. This includes utilizing substances like hemin, apolipoprotein A1 mimetic peptides, epoxyeicosatrienoic acid (EET), and peroxisome proliferator-activated receptor alpha (PPARα). However, we note that excessive or dysregulated expression or functioning of HO-1, coupled with other metabolic factors, may have detrimental effects, as observed in the neurovascular system [44].

## 4. GLUT-1 and GLUT-4: Essential Players in Myocardial Adaptation

The energy utilized by cardiomyocytes is generated from several interconnected metabolic pathways. Normally, about 70% of their energy comes from the beta-oxidation of fatty acids [45]. During this process, fatty acids are broken down into acetyl-CoA, which then enters the TCA cycle and the electron transport chain to generate ATP. However, under ischemic conditions, energy production shifts to anaerobic processes, and cardiomyocytes to rely on glycolysis as the preferred energy source to meet the increased myocardial energy demand. Interestingly, in embryonic and neonatal hearts, glycolysis is the primary source of energy production, whereas it shifts to beta-oxidation of fatty acids in fully developed adult hearts [46]. This transition suggests that glucose transporters play a crucial role not only in cardiac adaptation and protection but also in cardiomyocyte regeneration. They are essential not only in ischemic hearts but also in embryonic hearts, suggesting their significance in sustaining energy metabolism and facilitating cardiac function throughout different developmental stages.

**Figure 3.**
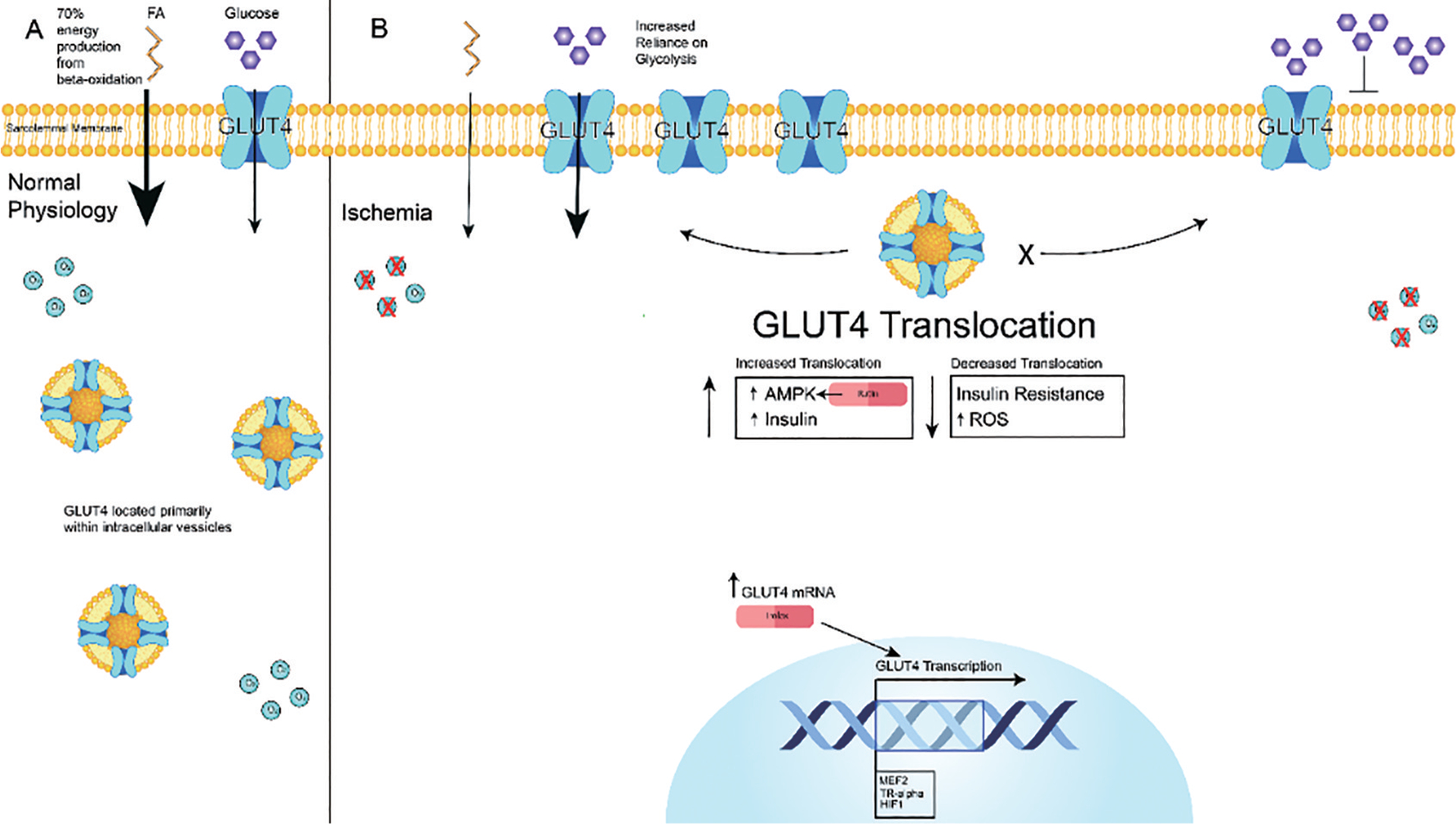
The role of GLUT-4 during ischemia. (**A**) During normal physiology cardiomyocytes primarily depend on energy production from beta-oxidation of fatty acids. The majority of GLUT-4 is contained within intracellular vesicles in the cytosol. (**B**) During ischemia cardiomyocytes switch from oxidative metabolism to glycolytic metabolism to preserve cardiac function under hypoxic conditions. In response GLUT-4 is translocated from intracellular vesicles to the cell membrane to facilitate cardiac glucose uptake and maintain adequate energy production. Diminished ability to upregulate GLUT-4 occurs with insulin resistance and excessive oxidative stress resulting in increased cardiac damage. Rutin is a flavanol which can increase GLUT-4 translocation via the activation of AMPK. Trolox, an antioxidant drug, can increase GLUT-4 translocation by fortifying the antioxidant system (as ROS inhibits GLUT-4 translocation) and by directly upregulating GLUT-4 transcription.

Glucose transporter-1 (GLUT-1) is responsible for basal glucose uptake, which operates independently of insulin. In contrast, GLUT-4 is insulin-dependent and primarily located in intracellular vesicles under basal conditions [47]. Studies have shown that GLUT-4 translocates to the cell membrane to facilitate cardiac glucose uptake in response to various stresses, including insulin stimulation and ischemia/hypoxia [45,48–50]. In cardiac muscle, myocyte enhancer factor-2 (MEF2) and thyroid hormone receptor alpha 1 (TR-alpha) are essential for the transcription of the Slc2a4 gene, which encodes GLUT-4 [51]. The HIF-1 promotes the expression of GLUT-4 mRNA during myocardial ischemia-reperfusion [18]. Ischemia/hypoxia induces significant translocation of GLUT-4 molecules to the plasma membrane of cardiomyocytes, while the combination of insulin with ischemia triggers an even more pronounced GLUT-4 translocation (Figure 3) [52]. The translocation of GLUT-4 stimulates glycolysis during ischemia, which is an important adaptive response to limit cardiac injury [53]. Notably, inhibition of phosphoinositide 3-kinases (PI3K) using wortmannin eliminated insulin-triggered GLUT-4 translocation and glucose uptake while leaving those induced by ischemia unaffected [54], suggesting disparate mechanisms between insulin and ischemia. Indeed, GLUT-4 translocation by either insulin or ischemia stimulated heart glycolysis by activating 6-phosphofructo-2-kinase (PFK-2), which increases the levels of fructose 2,6-bisphosphate, a glycolysis stimulator. However, PFK-2 activation by ischemia was mediated by AMPK [55], while PFK-2 activation by insulin was mediated by a different kinase[56], highlighting the intricate regulatory mechanisms governing glucose metabolism in the heart under these conditions. The expression level of GLUT-4 and its translocation to the sarcolemma of cardiomyocytes were found to be inhibited in experimental diabetic mice [57] and in humans with diabetes [58], suggesting a potential role of impaired GLUT-4 translocation in diabetic cardiac injury (Figure 3).

In response to dysregulated GLUT-4 translocation, cardiomyocytes activate compensatory mechanisms to mitigate apoptosis and prevent the progression of heart disease. One study revealed distinct responses of cardiomyocytes to chronic versus acute inhibition of GLUT-4 [45]. Acute inhibition resulted in more pronounced detrimental effects compared to chronic inhibition, primarily because cardiomyocytes were inadequately compensated for by increased GLUT-1 expression. While existing evidence strongly supports GLUT-4 as the primary glucose transporter for cardioprotection, this study suggests that GLUT-1 may serve as a promising compensatory mechanism when GLUT-4 function is compromised. This underscores the crucial role of cardioprotective processes and compensatory adaptations within the heart in response to alterations in glucose transporter function. Nevertheless, GLUT-1 play independent roles in the heart from that of GLUT-4. Fajardo et al. highlight the regenerative potential of embryonic hearts attributed to GLUT-1 upregulation. In their study, transgenic neonatal mice overexpressing GLUT-1 were subjected to cardiac injury, and their regenerative capacity was compared to that of wild-type mice. The results revealed that GLUT-1 transgenic mice exhibited lower levels of post-injury fibrosis compared to their wild-type counterparts. Further immunostaining demonstrated increased cardiomyocyte proliferation in the GLUT-1 transgenic mice [46]. These findings underscore the regenerative role of GLUT-1 in neonatal cardiomyocytes. However, it is important to note that cellular responses in postnatal cardiomyocyte development may differ from those in neonatal cardiomyocytes, which calls for further studies to elucidate the nuances of this shift [59].

Studies have established a strong correlation between hyperglycemia in diabetic patients and heightened levels of ROS alongside increased cell death [60]. This surge in ROS and oxidative stress was linked to diminished cellular translocation of GLUT-4, exacerbating cellular damage. To explore potential interventions, researchers administered Trolox, an antioxidant drug, to rat myocardial H9C2 cells exposed to high glucose conditions. They found that Trolox effectively counteracted the downregulation of GLUT-4 expression, fortified antioxidant system, and prevented DNA damage and mitochondrial-dependent apoptosis (Figure 3). These results highlight the promising therapeutic potential of Trolox in alleviating the detrimental effects of hyperglycemia-induced oxidative stress on cardiac cells [61].

Finally, exploring alternative pathways to induce GLUT-4 translocation to the sarcolemma of cardiomyocytes could yield significant therapeutic benefits. Notably, key regulators in the signal transduction pathway such as AMPK and Akt play crucial roles in facilitating GLUT4 translocation. By targeting these pathways, it may be possible to circumvent the insulin-dependent mechanism of GLUT-4 translocation, presenting a promising avenue for patients with diabetic cardiomyopathy.

## 5. HIF-1, HO-1, and GLUT-1/4 as Therapeutic Targets for Hypertension

Studies have indicated that HIF-1 is a robust target for controlling hypertension. For instance, dysregulated HIF-1 transcription can elevate ROS, predisposing individuals to hypertension [62]. The mechanism underlying HIF-1-dependent hypertension lies in the regulation of the carotid body chemosensory reflex, which senses arterial partial pressures of oxygen, carbon dioxide, and pH. Studies indicate a pharmacogenomic aspect of HIF-1, where partial deficiency of the gene may reduce the carotid body’s response to hypoxia detection, potentially slowing the development of hypertension. Indeed, mice with heterozygous HIF-1α deficiency exhibited a notable absence of hypertension induced by chronic intermittent hypoxia when compared to gender-matched wild-type littermates [63]

Moreover, chronic stimulation of the carotid body results in increased sympathetic activity, leading to heightened release of catecholamines and subsequent vasoconstriction, thereby contributing to elevated blood pressure [64]. Several drugs show promise as potential treatments for HIF-1-induced hypertension [65], including topotecan [66], celastramycin [67], and YC-1 [68]. These medications attenuate pulmonary arterial hypertension by inhibiting HIF-1 at various levels, including transcriptional and translational mechanisms [65]. Despite the diverse therapeutic options, including those investigated in mouse studies for pulmonary hypertension, clinical trials for these drugs have not yet been conducted. Further exploration of these pharmacological interventions through human trials holds the potential to advance strategies for managing hypertension.

The modulation of hypertension by HO-1 is a topic of intrigue, although its precise mechanism remains elusive. Through its metabolites generated via heme-releasing processes, HO-1 exerts multifaceted effects on blood flow. Notably, the conversion of biliverdin to bilirubin by biliverdin reductase holds significance, as bilirubin plays a crucial role in maintaining cardiovascular health and blood pressure [69]. Elevated bilirubin levels, alongside CO production, indirectly inhibit the angiotensin type 2 receptor (ANG II), thereby lowering blood pressure. This mechanism counters the vasoconstrictive effects of ANG II, primarily mediated by ROS production via NADPH oxidase.

Prevailing evidence suggests that targeting HO-1 induction may be an effective therapeutic strategy for controlling hypertension which is unresponsive to conventional antihypertensive medications. The conjugation of bilirubin and CO, key factors produced by HO-1, plays a pivotal role in hypertension prevention. Also, HO-1 can be induced by natural products such as curcumin, flavonoids, isothiocyanates and organosulfur compounds which may have antihypertensive effect [70]. Indeed, curcumin lowers blood pressure in many models of experimental hypertension although the specific role of HO-1 induction in these effects is not determined [71]. Some hypertensive patients may benefit from dose-dependent HO-1 agonists, such as Oxyberberine [69], which further stimulate their production. It’s crucial to note potential adverse effects, including an increased risk of jaundice and scleral icterus, particularly in patients with a history of hepatocellular injuries or elevated serum bilirubin levels. Although research on HO-1 agonists and their efficacy in treating chronic hypertension is limited, they hold promise as a potential therapeutic target for patients who have not responded adequately to multiple lines of antihypertensive agents. Further exploration of HO-1 modulation could unveil novel therapeutic avenues for managing hypertension and related cardiovascular complications.

**Figure 4.**
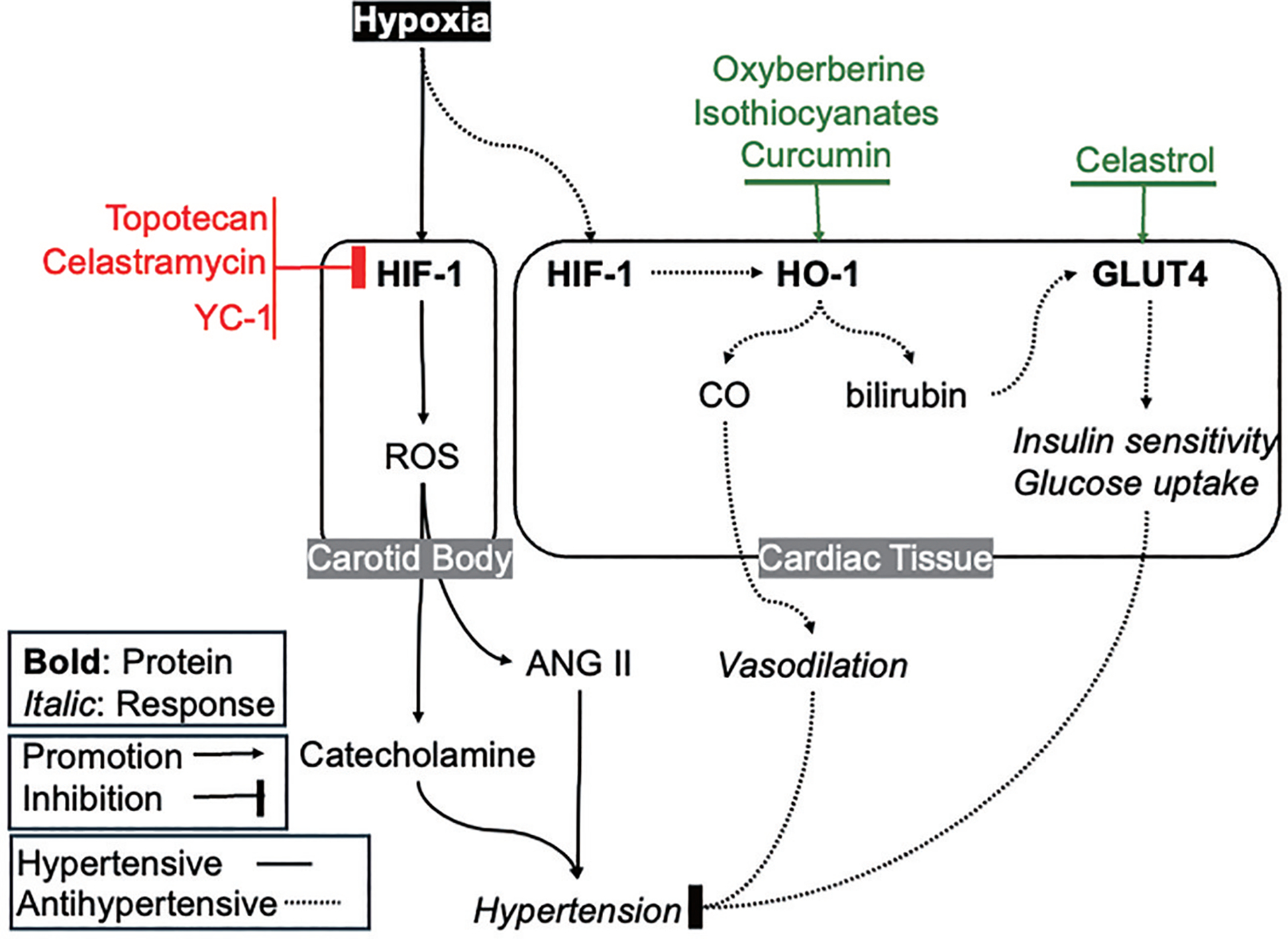
Targeting HIF-1, HO-1, and GLUT-4 for Hypertension Therapy.

A decline in insulin-dependent GLUT-4 transport can contribute to hypertension, as these transporters are involved in the regulation of cardiac contractility [72]. Thus, there exists a significant interplay between myocardial insulin resistance in diabetic patients and the onset of hypertension. Moreover, beyond insulin resistance and diabetes, obesity poses as an additional risk factor. In an experimental study, the medication Celastrol was administered to obese mice, resulting in reduced fat intake by targeting galanin receptors in the hypothalamus [73]. Another study reported that Celastrol can enhance glucose uptake via GLUT-4 transporters to initiate a cascade of essential metabolic processes vital for cardiac tissue. Such novel medications hold potential for addressing a constellation of metabolic health issues including obesity, hyperlipidemia, diabetes mellitus and hypertension/ cardiac disease (Figure 4).

## 6. HIF-1, HO-1, and GLUT-1/4 as Therapeutic Targets for Hyperlipidemia/Atherosclerosis

Atherosclerosis is a major risk factor of cardiovascular disease, particularly coronary artery disease, precipitating systemic vasoconstriction through the accumulation of lipid deposits within vascular walls, consequently impeding blood flow. This condition increases the risk of hemorrhage as plaques, composed of liquid deposits, detach from vessel walls, potentially culminating in significant clot formation and subsequent myocardial infarction. Patients presenting with developing or fully formed atherosclerosis typically exhibit elevated levels of low-density lipoprotein (LDL) and reduced levels of high-density lipoprotein (HDL) in their serum.

Aberrant HIF-1 expression results in increased production of ROS levels, triggering the development of hypertension. The primary function of HIF-1 is to regulate the response of the carotid body to oxygen levels. Compounds that inhibit HIF-1 activity may potentially mitigate the risk of hypertension. In cardiomyocytes, HIF-1 and its downstream mediator HO-1, generate carbon monoxide and bilirubin which function to maintain the cardiovascular health and reduces blood pressure by counteracting the effects of angiotensin II. Natural substances that induce HO-1 expression may be effective in preventing hypertension. Data indicate that GLUT-4 maintains normal blood pressure and enhances cardiac glucose uptake. Reduced activity of GLUT-4 may contribute to hypertension, suggesting a correlation between insulin resistance, obesity, diabetes, and elevated blood pressure.

**Figure 5.**
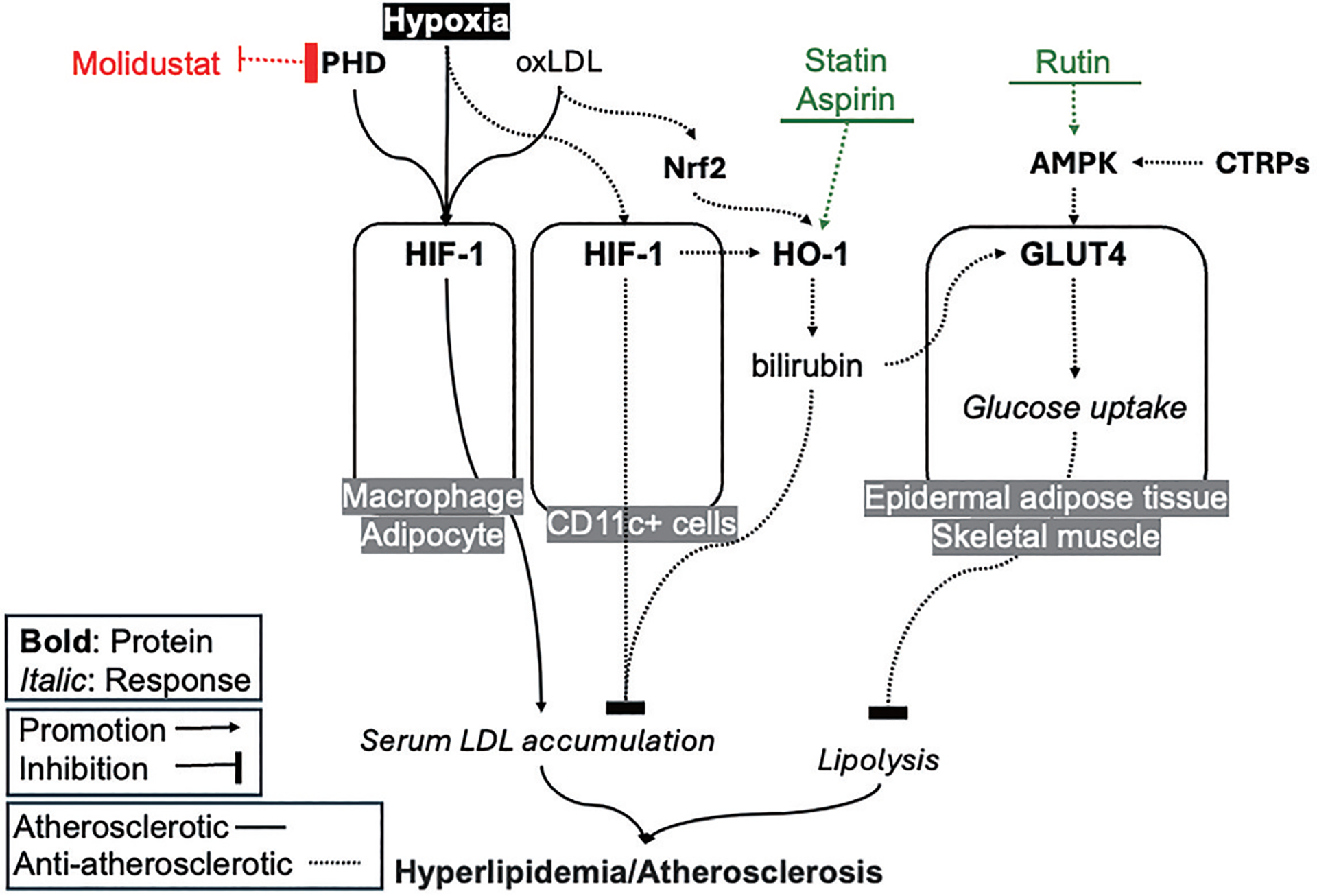
HIF-1, HO-1, and GLUT-4 as Therapeutic Targets for Hyperlipidemia/Atherosclerosis.

The role of HIF-1α in atherosclerosis is complicated as both detrimental and protective effects have been reported (Figure 5) [74]. The stabilization of HIF-1α is often detected in hypoxic environments and in the presence of high serum levels of oxidized LDL, particularly in macrophage exposed to LDL within atherosclerotic lesions [74,75]. The stabilization of HIF-1α may promote or worsen the atherosclerotic status in high-risk patients by initiating and promoting the formation of foam cells, endothelial cell dysfunction, apoptosis, stimulating inflammation and angiogenesis [74–77]. In mice, deletion of HIF-1α in macrophages or adipocytes inhibited the occurrence of atherosclerosis [77,78], supporting a pathogenetic role of HIF-1α in atherosclerosis. However, deletion of HIF-1α in CD11c+ antigen-presenting cells accelerated atherosclerotic plaque formation and increased lesional T-cell infiltrates [79], highlighting its protective role against atherosclerosis (Figure 5). Together, these studies suggest the dual role of HIF-1α in atherosclerosis; whether HIF-1α is detrimental and protective is likely influenced by cell type specificity and context dependency. Despite the observed dichotomy in the role of HIF-1 in experimental atherosclerosis, both genetic and pharmacological inhibition of prolyl hydroxylase domain enzymes (PHD) have shown its ability to inhibit atherosclerosis development in high-fat-diet-fed LDL receptor-deficient mice (Figure 5) [80]. Further investigation is required to fully understand the specific mechanisms underlying the dual effects of HIF-1α and their implications for the progression of atherosclerosis.

Atherosclerosis is caused by plaque buildup that contains low-density lipoprotein (LDL) and macrophages inside the arteries. Whether hypoxia- and oxidized LDL (oxLDL)-induced HIF-1α is harmful or beneficial in atherosclerosis depends on the specificity of cell type and the context in which it occurs. HO-1 plays a crucial role in protecting against progressive atherosclerosis, while the Nrf 2-mediated regulation of HO-1 influences the accumulation of LDL. Upregulating the expression of GLUT4 can help mitigate the harmful metabolic effects of hyperlipidemia and obesity. C1q/TNF-α related proteins (CTRPs) and AMPK can potentially be used as a therapeutic target to activate GLUT4 and improve glucose uptake.

Notably, HO-1 can prevent the progression of atherosclerosis, with oxidized LDL serving as a potential inducer of HO-1 [81]. On one hand, a deficiency in HO-1 is linked to hyperlipidemia and the formation of fatty streaks and fibrous plaques in the human aorta [82]. On the other hand, studies have shown that over-the-counter medications such as aspirin, as well as drugs like statins, can induce HO-1, which aids in preventing the progression of atherosclerosis [83]. The metabolite of HO-1, bilirubin, functions as an exceptional antioxidant, actively mitigating LDL accumulation by inhibiting its oxidation, thereby preventing complications like atherosclerosis [84,85]. Intriguingly, there exists Nrf 2-mediated regulation of HO-1 in antioxidant activity, influencing LDL accumulation (Figure 5). For instance, Nrf 2/HO-1 signaling facilitates the release of antioxidant metabolites, such as bilirubin, in response to oxidized LDL, consequently diminishing ROS production [83, 86]. However, evidence suggests that, via a distinct metabolic pathway, Nrf 2 may potentially contribute to atherosclerosis by promoting liver lipogenesis of non-HDL metabolites [83], despite the lack of mechanistic details regarding the regulatory signals driving increased liver lipogenesis. Further research is needed to fully understand the relationship between Nrf 2/HO-1, hyperlipidemia and atherosclerosis.

Individuals with hyperlipidemia and obesity could potentially benefit from upregulated GLUT4 expression, as this gene enhances glucose uptake. By augmenting GLUT4 expression and enhancing glucose uptake, the adverse metabolic effects associated with hyperlipidemia and obesity can be avoided, suggesting that this approach may offer potential therapeutic avenues for managing these conditions (Figure 5). Epicardial adipose tissue has been proposed as an independent cardiometabolic risk factor for coronary artery disease (CAD) [87]. Studies have found reduced GLUT4 expression in epicardial adipose tissue [88,89] and skeletal muscle [90] among obese patients with CAD and insulin resistance, suggesting a potential role of GLUT4 in the development of CAD and its associated metabolic abnormalities. These observations underscore the significance of investigating the interplay and implications between adipocyte secretions and glucose uptake in the context of heart disease. Numerous studies have explored the potential of anti-adipogenic medications in this regard. For instance, research on Moringa oleifera has identified a compound called Rutin, which reduces adipogenesis and consequent lipid accumulation, leading to increased glucose uptake through AMPK/GLUT-4 pathways (Figure 5) [91]. Further research into the role of AMPK as a potential therapeutic target for cardiomyopathy, diabetes, and hyperlipidemia is warranted, given its presence in the signal transduction pathway that ultimately leads to the translocation of GLUT4 to the sarcolemma[92]. Of note, a study found that C1q/TNF- α related proteins (CTRPs) stimulated AMPK phosphorylation and led to increased GLUT4 translocation[93]. Thus, investigating these alternative pathways to enhance glucose transport into cardiomyocytes could potentially facilitate the metabolic processes necessary to mitigate ischemic heart disease.

## 7. Clinical Implications and Future Research

This review explores the roles, regulation, and interplay of key cardioprotective genes HIF-1, HO-1, GLUT-1, and GLUT-4, with a specific focus on their significance in the context of ischemic stress and associated conditions such as diabetes, hyperlipidemia, and hypertension. Understanding the mechanisms that mediate the cardioprotective effects of these pathways provides a crucial foundation for developing targeted therapies that transcend mere symptom management. HIF-1, with its pivotal role in angiogenesis, inflammation, and immune function, holds promise in drug development to enhance cardioprotection and modulate the inflammatory response. Similarly, HO-1, with its capacity to modulate oxidative stress, presents an opportunity for drug development to manage ischemia/reperfusion injury and promote recovery, potentially contributing to cell repair and tissue regeneration. Moreover, GLUT-1 and GLUT-4, which modulate metabolic adaptations, offer potential avenues for drug development, particularly in scenarios where energy production pathways are compromised, such as in ischemic conditions.

Harnessing these pathways and targets through drug development holds promise for addressing the underlying mechanisms of cardiovascular diseases, laying the foundation for developing more effective therapeutic approaches. However, unresolved issues pose challenges to this endeavor. Understanding how these pathways and mechanisms coordinate to provide optimal cardioprotection remains a significant challenge, necessitating further investigation. Additionally, targeting upstream and downstream players within the signal transduction pathways of these genes presents a promising avenue for future research. Further studies, laboratory and clinical, are advocated to accelerate the translation of these discoveries into practical drug interventions for cardiovascular diseases, aiming to improve patient outcomes and quality of life.

In summary, the intricate and coordinated roles of HIF-1, HO-1, GLUT-1, and GLUT-4 in cardioprotection unveil potential avenues for drug development. Targeting these cardioprotective genes could offer innovative therapeutic strategies for conditions such as ischemic heart disease, diabetes, hyperlipidemia/atherosclerosis, and beyond.

## Abbreviations:

HIF-1: Hypoxia-inducible factor-1
HO-1: heme oxygenase-1
GLUT-1/4: Glucose transporter-1/4
ARNT: arylhydrocarbon receptor nuclear translocator
PHD1/2/3: Prolyl hydroxylase domain proteins 1/2/3
pVHL: von-Hippel-Lindau protein
HRE: hypoxia responsive element
VEGF: vascular endothelial growth factor
EGF: Epidermal growth factor
TIMPs: tissue inhibitors of metalloproteinases
EGFR: epidermal growth factor receptor
PI3K: phosphoinositide-3-kinase
mTOR: mechanistic target of rapamycin
eNOS: endothelial nitric oxide synthase
CO: carbon monoxide
ROS: reactive oxygen species
ARE/EpRE: antioxidant/electrophile response elements
NF-kB: nuclear factor-kappa B
Nrf 2: nuclear factor erythroid 2-related factor 2
AP-1: activator protein-1
Bcl2: B-cell lymphoma 2
CK-MB: creatinine kinase MB
mtDNA: mitochondrial DNA
STZ: streptozotocin
EET: epoxyeicosatrienoic acid
PPARα: peroxisome proliferator-activated receptor alpha
MEF2: myocyte enhancer factor-2
TR-alpha: thyroid hormone receptor alpha 1
PFK-2: 6-phosphofructo-2-kinase
AMPK: AMP-activated protein kinase
ANG-II: Angiotensin 2
LDL: low-density lipoprotein
HDL: reduced levels of high-density lipoprotein
CAD: coronary artery disease
CTRPs: C1q/TNF-α related proteins
